# Targeted Verifiable Subcision for Management of Cellulite on the Buttock and Thigh: Incorporation Into Real-World Lower Body Rejuvenation Approaches

**DOI:** 10.1093/asjof/ojae050

**Published:** 2024-07-03

**Authors:** Johnny Franco, M Brad Calobrace, Matthew R Schulman

## Abstract

**Background:**

Cellulite affects 80% to 98% of postpubertal females, and most patients with the condition are bothered by it. Targeted Verifiable Subcision (TVS; Avéli; Revelle Aesthetics, Inc., Mountain View, CA) is a minimally invasive mechanical subcision device that reduces the appearance of cellulite dimples in the buttocks and thighs. Although clinical trials have demonstrated efficacy, information on real-world surgical and nonsurgical approaches to managing cellulite in buttocks and thighs is needed.

**Objectives:**

To describe how TVS is being used by the authors to improve patient outcomes in the lower body in both surgical and nonsurgical settings.

**Methods:**

Each of the authors described their current practices using TVS for lower body rejuvenation. Author practices have varied proportions of surgical vs nonsurgical cases (from 10% to 100% surgical cases) and a variety of focuses, ranging from primarily facial aesthetics to a near exclusive focus on body contouring.

**Results:**

The authors' diverse approaches to integrating TVS into clinical practice are detailed, along with pearls of clinical success. TVS is discussed as a companion treatment for surgical procedures, while the patient is already under anesthesia, as well as a part of a nonsurgical approach, where the local anesthesia required for TVS can make additional energy-based procedures more comfortable for the patient. The authors provide several examples and include considerations for optimal timing and ordering of treatments.

**Conclusions:**

TVS may become a valuable addition to the treatment armamentarium for surgical or nonsurgical buttock and thigh rejuvenation and can be integrated into surgical and nonsurgical workflows.

**Level of Evidence: 4:**

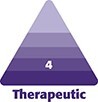

Lower body rejuvenation has become an integral part of modern aesthetics; however, the lower body is a complex area to treat, often requiring a combination of multiple treatments to address skin laxity, volume, and contour issues. For rejuvenation of the buttocks and thighs, the art of aesthetic transformation lies in understanding patients’ priorities, assessing patients carefully, and helping patients understand the limitations of individual treatments.

For the buttocks and thighs, patient experience hinges upon improvement in *all* key issues important to them. Cellulite affects as many as 80% to 98% of postpubertal females across all races and ethnicities: most patients who have the condition are embarrassed and bothered by it,^1-4^ and resolution of cellulite can improve both quality of life and self-esteem.^4^ Although very few patients present specifically seeking treatment for cellulite, in the authors’ experience, its management is central to a positive patient experience with lower body rejuvenation: if left unresolved, patient satisfaction with other procedures will likely decrease. Conversely, even if patients do not initially mention cellulite as a key concern, they are often bothered by residual dimples after undergoing procedures such as gluteal augmentation or treatments to address skin laxity, even when these dimples were not noticed or bothersome prior to body contouring treatments. Most patients will refer to a variety of concerns on the buttocks and legs as “cellulite,” and it is imperative that the treatment provider guide patients through the various pathologies that contribute to overall global appearance, including skin laxity, volume loss, and cellulite. The correct diagnosis will lead to the correct treatment. In 2022, there were over 700,000 surgical lower body procedures performed^5^ and 400,000 nonsurgical body contouring procedures in the United States. Although the number of patients seeking lower extremity rejuvenation is expected to grow, owing to the popularity of new, revolutionary weight-loss medications, the number of current procedures alone represents an enormous opportunity to further improve patient experience and outcomes by treating cellulite.

In 2022, the FDA cleared Targeted Verifiable Subcision (TVS; Avéli; Revelle Aesthetics, Inc., Mountain View, CA) for the long-term reduction of cellulite in buttocks and thighs.^6^ TVS is a minimally invasive subcision device that permits mechanical, focal release of the fibrous septal bands that cause cellulite. The device also permits verification of the contribution of septa to a cellulite dimple prior to cleavage as well as complete treatment following subcision. Its minimally invasive nature, coupled with focal (and verifiable) action underpins TVS predictability and is, in part, why it can feasibly be combined with other procedures.

As with many aesthetic devices, the data generated to support regulatory clearance do not provide complete evidence for how TVS can be used in real-world clinical practice, especially in combination with other procedures. This type of information is important, as most patients, whether being treated on the face or body, require multiple, mechanistically distinct interventions. In this study, the authors detail their own experiences incorporating TVS into their current procedures and office workflows, including the diagnosis of cellulite and compounding issues, as well as surgical and nonsurgical treatment approaches.

The authors' purpose is to describe how TVS for the management of cellulite has been incorporated into surgical and nonsurgical workflows in their clinics. The intention of this article is to share with the broader aesthetics community how TVS can improve patient experience with lower body rejuvenation in a manner that is efficient and economically viable for the clinic.

## METHODS

Each of the 3 authors is highly experienced in the management of cellulite using multiple technologies, including TVS. Together, they have treated over 250 patients with TVS, both as a stand-alone therapy as well as with other modalities as part of a surgical or nonsurgical approach. The authors have varying proportions of surgical and nonsurgical cases in their practice, ranging from 10% to 100% surgical cases, as well as a variety of different practice focuses, ranging from a primary focus on facial aesthetics to an almost exclusive focus on body contouring. Their clinical experience is the basis for the information presented here. Because this is not a prospective or a retrospective study, IRB approval was not required. Each patient provided written informed consent prior to the procedure and has provided written consent to have their photographs published. All patients were treated in accordance with the principles outlined in the Declaration of Helsinki.

### Treatment With the Targeted Verifiable Subcision Device

Following injection of the tumescent solution (Video 1), one of several anesthesia options, the TVS 15 cm probe is advanced through a single or small number of transcutaneous incisions, in the subcutaneous layer, to the dimples to be treated. A light source at the tip of the probe assists the clinician in tracking the device's path and precisely confirms probe depth, ensuring reproducible results and safety. Once the probe is in the proper position in relation to the cellulite dimple, a small hook is deployed, which is then used to verify and then release septal bands contributing to the dimple. The hook is then passed under the dimple to visually verify the release of the septal band causing the dimple formation. An example of treatment in the buttocks is shown in Video 2, and that in the thighs in Video 3. Like other mechanical subcision–based methods, the treatment effect is predicted to be permanent as the bands have never been shown to re-form.^7,8^ For TVS, 12-month data support the durability of treatment effect, which is maintained from Month 3 onward, so the treatment is most often presented as a 1-time, long-lasting, if not permanent treatment.^9^ The treatment is technically straightforward and can be delegated in the surgical and nonsurgical setting.

## RESULTS

**Figure 1.**
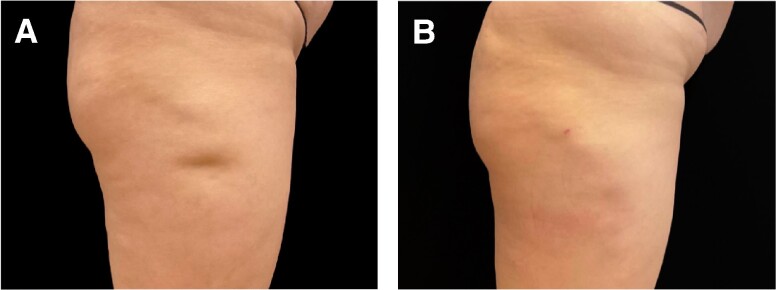
A 48-year-old female at baseline (A) and 4 weeks after treatment with Targeted Verifiable Subcision (B) for isolated cellulite in the buttocks and thighs. Twenty-seven total depressions treated. Aveli immediately followed by 1 treatment of radio frequency microneedling to thighs (image courtesy of Dr Franco).

**Figure 2.**
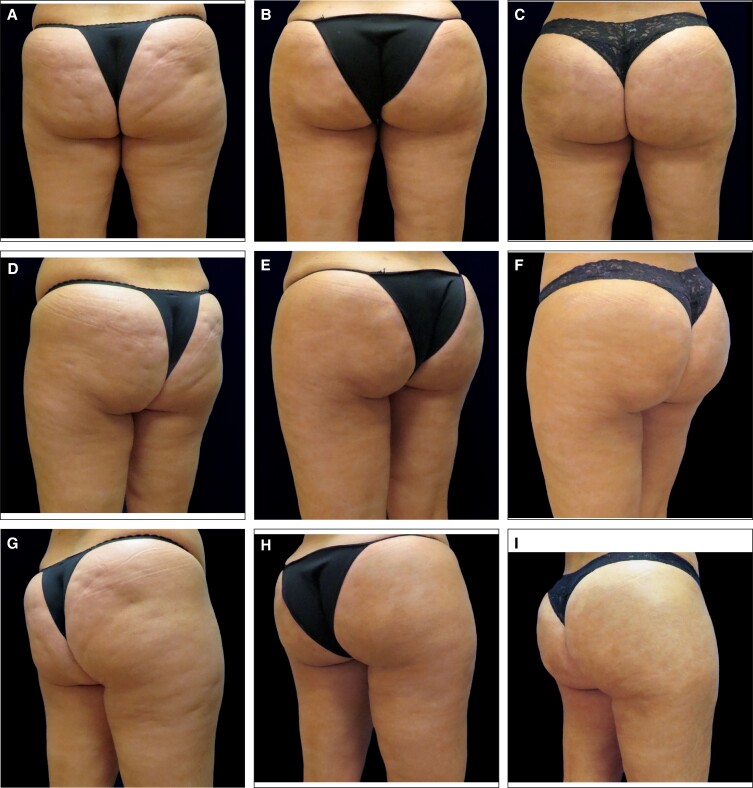
A 52-year-old female patient at baseline (A, D, G), 3 months after (B, E, H), and 1 year after (C, F, I) removal of 5 L (5000 cc) of from her abdomen, flanks, and back using liposuction and injection of 1350 cc of fat into each buttock and hip. Immediately following fat transfer, the patient was treated with Targeted Verifiable Subcision to resolve her cellulite dimples. Note, the improved shape of the torso from the liposuction and increased volume and shape of the buttocks and hips from the fat transfer as well as the reduced appearance of cellulite (image courtesy of Dr Schulman).

**Figure 3.**
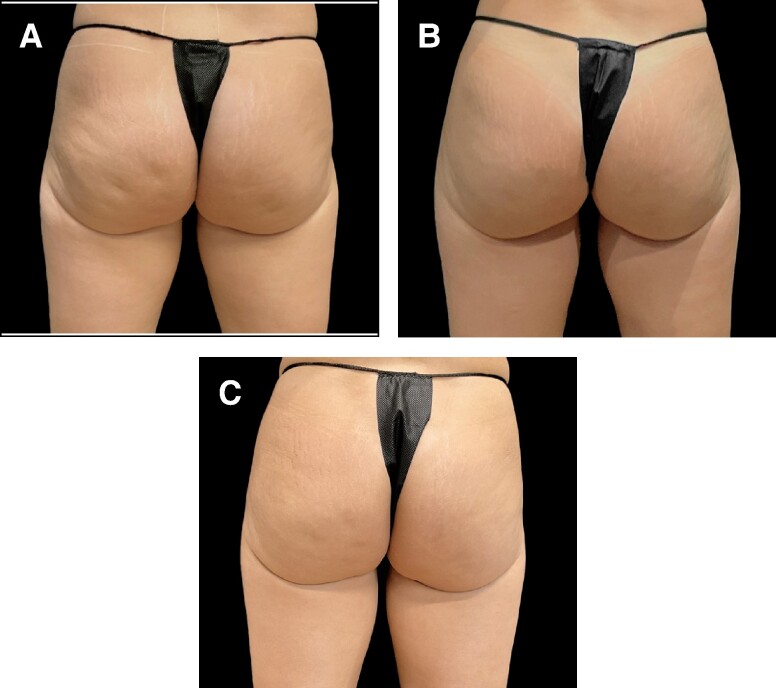
A 47-year-old female at baseline (A) and 3 months following treatment with nonsurgical fat reduction to the lower flanks, Targeted Verifiable Subcision, and microneedling with radiofrequency to the buttocks and thighs (series of 3, spaced 4 weeks apart) followed by 20 syringes of hyperdilute CaHA to the buttocks (B). The patient is also shown 12 months following treatment (C; image courtesy of Dr Franco).

**Figure 4.**
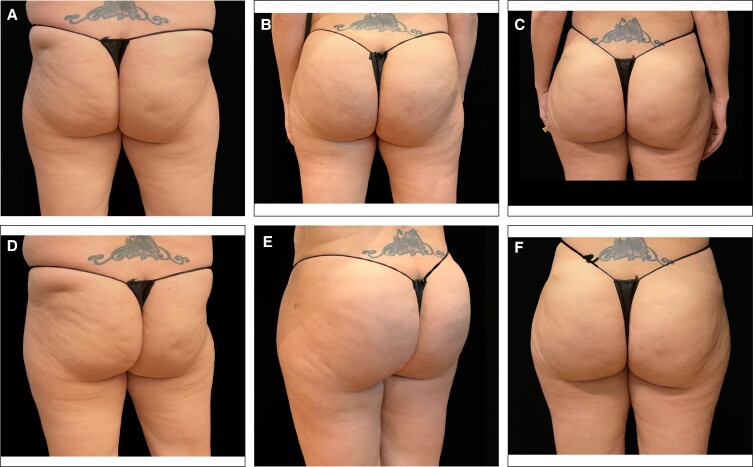
A 46-year-old female at baseline (A, D), 3 months (12 weeks) after Aveli and 6 weeks after Brazilian butt lift (B, E), and 9 months following Aveli (C, F). Liposuction was done to abdomen back and flanks and then 1300 cc of fat was transferred per side to the buttocks. Thirty total depressions were treated (image courtesy of Dr Franco).

In the following sections, the authors discuss how they have integrated TVS into their clinical practices. Case studies illustrating results that can be achieved using TVS are shown in Figures 1 to 4.

## DISCUSSION

### Differential Diagnosis and Treatment Planning

In the authors' experience, very few patients, who are treated for cellulite, can be optimally managed by only releasing isolated dimples; although there are some instances where the patient is only bothered by a small number of dimples (Figure 1). More often, the patient must be treated with a range of devices/procedures. Cellulite dimpling very rarely occurs in isolation, and most often patients also present with skin laxity and volume deficits that can exacerbate the appearance of dimples and should ideally be managed along with cellulite. Although education around the contribution of laxity and cellulite on overall aesthetic is important, patients are not generally interested in differentiating the impact of separate pathologies or treatments on their global appearance. Rather, they count on the expertise of their treating clinician to suggest treatment programs which will improve the global appearance of their buttocks and thighs. The process and time needed for any given combination must be clearly defined and mapped out and patients must clear results, starting from the initial session, in order to maintain interest in continuing the process of lower body rejuvenation.

### Author Integration of Targeted Verifiable Subcision Into Clinical Workflows

Combining TVS with other nonsurgical or surgical modalities into treatment packages for the buttocks and/or thighs makes treatment more efficient for the patient and the practice and permits the clinician to more easily recommend a treatment course that will lead to global improvement with which the patient will be satisfied.^10^ In general, ordering and combining treatments should always be guided by patient priorities as well as treatment mechanism, side effects, and time to peak effect. The fact that TVS action takes place within a small area, within a single tissue plane, and necessitates a small number of transcutaneous incisions makes it amenable to combination treatment. Several examples are provided below.

### Targeted Verifiable Subcision and Surgical Gluteal Augmentation

Because of the minimal additional procedure time and risk associated with TVS above and beyond those of gluteal augmentation, all of the authors present TVS as a 1-time, optional add-on treatment for this procedure. Fat grafting is not able to resolve cellulite dimples, so treatment in combination with augmentation is important for achieving an optimal improvement. The examples are presented in Figures 2 to 4 along with key learnings from each of the authors’ practices.

#### During Surgery

When TVS is carried out along with fat transfer (Figure 2), the patient is under anesthesia, and the procedure can be performed quickly. There are several investigator-initiated studies in development to delineate best practice for simultaneous cellulite treatment and buttock augmentation, which will be important for determining the ideal approach, as there are benefits to managing cellulite both before and after fat grafting. Although cellulite dimples can be released prior to fat transfer to the buttock, as added volume in the buttock can make treating the cellulite challenging. TVS can also be used after fat grafting, when added fat can provide added tension to the tissue and permit dimples to be more easily visualized and released. Irrespective of treatment order, the following precautions are important for simultaneous fat transfer and cellulite treatment.

#### Respecting Tissue Plans

Because fat grafting is done in a deeper plane than the more superficial TVS, fat placement should not be disrupted by TVS. In the authors' clinical experience, respecting tissue planes can improve the overall outcome and decrease the risk of complications. In one of the author's practice, retrospective data in over 30 patients suggest that contour deformities, or “blow-out fractures,” can be avoided when fat transfer is completed in the deep fat plane above the muscle fascia and cellulite treatments are performed in the superficial plane using the light-guided TVS system. Ultrasound-guided fat transfer placement can aid in precisely placing the fat exclusively in the intended planes, and the light on the TVS device can be used to ensure superficial location.

#### Buttock Volume

When combining fat transfer and cellulite treatment on the same day, it is important to be cognizant of the stress put on the skin by the larger volume of fat transfer and to exercise caution.

#### Energy Devices

When combining energy devices, fat transfer, and cellulite treatment in a single session (Figure 3), extreme caution must be taken until further protocols have been evaluated for safety.

#### Judicious Treatment

When planning the number of individual dimples to treat, it is important not to select too many dimples close together as doing so can create a lake effect or blow-out fracture. Remember, the septal network is also responsible for maintaining the shape of the buttocks, and excessive release of the network can cause local flattening or encourage fat protrusion, especially when combined with fat transfer procedures. Because TVS is effective and reproducible, there is no need to treat it excessively or broadly. Instead, moderate, precise application of the technology is safest and leads to the best results. Typically, the authors will avoid treating any dimples within 2 cm of the gluteal fold.

#### After Surgical Lower Body Procedures

Approximately, 8 to 12 weeks after gluteal augmentation, TVS can be used as a stand-alone treatment to resolve cellulite dimples. It is important to wait several weeks after the initial augmentation to maximize fat retention and to permit resolution of normal edema (which can make it difficult to identify areas that need to be treated with TVS). This timing for TVS is most common when patients have had gluteal augmentation but have not had cellulite treated at the time of the procedure and would like to improve their aesthetic appearance to the best degree possible (Figure 4).

Patients with severe skin laxity requiring surgical removal of skin, such as a buttock lift or circumferential lift to remove excess skin, should be treated for laxity with, or prior to, TVS. In the authors’ experience, TVS can be performed as part of a surgical lift (immediately after the lift is performed).

#### Before Surgery

Pretreatment with TVS, 3 weeks or more ahead of the grafting procedure, can allow for more aggressive fat transfer. Furthermore, the potential for decreased complications is being explored. This timing is also beneficial for patients who may not be able to have surgery for a period of time for a variety of factors, as the patients are able to see clear, immediate improvements in the overall appearance of their buttocks as part of their lower body rejuvenation plan. Immediate resolution of cellulite, which is often a key patient concern, can be rewarding for patients and motivate them to continue with treatment.

### Targeted Verifiable Subcision and Nonsurgical Procedures

Although not all combinations are specifically detailed here, nonsurgical approaches to the buttocks and thighs should include attention to cellulite, skin laxity, diminished muscle tone, and/or depleted volume. Approaches for combining TVS with nonsurgical skin modalities for laxity are discussed in the following, and multiple examples of nonsurgical treatment plans used by the authors are presented in Table 1.

**Table 1.** Examples of Nonsurgical Treatment Plans

Examples of nonsurgical treatment plans using TVS
*Example 1* Initial treatment (same day) RF microneedling TVS administered Biostimulatory filler (hyperdilute CaHA, PLLA) Second treatment (6-8 weeks later) RF microneedling Biostimulatory filler (hyperdilute PLLAs or CaHA) Filler to improve lateral volume (ie, hip dips) Third treatment RF microneedling Biostimulatory filler (hyperdilute PLLAs or CaHA) Filler to improve volume (if still needed)
*Example 2* Initial treatment Hyperdilute CaHA (1st of 2 rounds) RF microneedling or RF helium-plasma with TVS Second treatment (6-8 weeks later) RF microneedling Hyperdilute CaHA (2nd of 2 rounds) Third treatment (6-8 weeks later) RF microneedling
*Example 3* Initial treatment RF microneedling (note—it is rare to only do 1 treatment of RF microneedling) TVS Filler

PLLA, Poly-L-Lactc Acid; TVS, Targeted Verifiable Subcision.

### Nonsurgical Skin Tightening

Laxity is one of the most common issues that is treated with cellulite in the nonsurgical setting. Patients with mild-to-moderate skin laxity who do not need surgical excision can be treated with a combination of energy- or ultrasound-based skin-tightening treatments in combination with TVS. Because TVS uses local anesthesia (Video 1), combining TVS with energy-based treatments such as radio frequency (RF) microneedling can be very efficient and comfortable for patients, with the combined usage also minimizing the number of treatment sessions needed. Because patients with moderate skin laxity often need 6 to 8 RF microneedling treatments to reach their desired goals, this is an important aspect of care. Although patients with minimal skin laxity have more options in terms of treatment order, many of the authors elect to do simultaneous TVS and RF treatment for these patients, as the results can be dramatic and the local tumescent creates a very comfortable experience for the patients. Patients with mild skin laxity often need between 3 and 4 RF microneedling treatments to reach their desired goals. When injecting local anesthesia, it is important to take time and be gentle. Otherwise, there is an increased risk of bruising and swelling. Most often, premedication is not required; however, a very small number of patients in the authors' practices have been given 50% nitrous oxide and oxygen gas.

Biostimulatory agents such as hyperdilute CaHA and Poly-L-lactic acid fillers are also an important tool for managing laxity,^11^ and when used in combination with energy-based devices, they can have a synergistic effect.^12^ In the setting of weight loss, where so many patients will have mild-to-moderate skin laxity, this is particularly important. Laxity is most often managed over the course of multiple sessions for skin tightening (up to 3 rounds of treatment spaced 4 months apart) and TVS is carried out at various time points ranging from at the initial treatment to ∼4 months following the final treatment depending on the priorities of the patient and degree of skin laxity.

#### Safety and Postprocedure Care

Experiences with past cellulite devices have led most practitioners and patients to be skeptical and cautious due to concerns around bruising and pigmentation; however, in the authors' experience, the bruising with TVS is minimal when compared with previous treatments. For TVS, the mechanism of cellulite treatment is mechanical and directly addresses the septa, which causes cellulite dimpling, but unlike subcision methods where the skin is broken at every dimple, TVS requires a minimal number of incisions, thereby eliminating additional mechanical trauma to the skin at each dimple such as suction or passage of a needle through the skin. In addition, the action on the septal network within the superficial plane is direct and focal, without disruption of deeper septal bands or vessels. In the authors' experience, patients experience minimal bruising, and both bruising and swelling are generally mild and self-limiting and generally dissipate within 2 to 3 weeks. Logistically, this is an important feature of treatment as TVS does not have the same seasonal restriction (ie, only treating patients in the “off season”) as previous technologies. In addition, the authors do not find that additional interventions are needed to clear bruising or resolve hemosiderin staining.

Prior to treatment, patients are advised to avoid aspirin, ibuprofen, alcohol, and other substances that increase the risk of bruising for 10 days prior to treatment. To further reduce the risk of bruising, epinephrine (1:100,000) can be added to local anesthetic solution, and tranexamic acid (0.5 g in 500 cc of solution) injected locally. Of the 68 patients who participated in the 12-month study, 1 (1.5% of patients) had hyperpigmentation of the skin at 6 months, the remainder of adverse events (AEs) were resolved by this time. At ≤3 months, the most common AEs were ecchymosis/bruising (*n* = 59, 86.8%), tenderness (*n* = 35, 51.5%), and pain (*n* = 26, 38.2%); most AEs were mild.

When patients undergo a Brazilian Butt Lift (BBL) and TVS, the compression garments used for BBL are sufficient to mitigate bruising and swelling, and there are no additional post procedure measures needed for TVS. If TVS is carried out as an isolated procedure, compression garments should be worn for 5 days, with lighter compression for another 10 days (for these 10 days, the light compression afforded by leggings, bicycle shorts, or consumer-grade light compression garments is generally sufficient).

The limitations of this article are inherent in the methodology. The information presented here is based on the authors’ experiences and expertise, which, while certainly valuable, are not equivalent to clinical evidence. In order to make evidence-based recommendations, a study of specific approaches with a control group and validated measurements of cellulite, along with formal safety monitoring, would be needed. For any study of combination treatments, long-term data would be ideal for determining the best course of action. Realistically, for assessing best practices for treatment ordering and outcomes, a real-world study or retrospective analysis could provide some insights into the safety of specific approaches and inform clinical care. Nevertheless, until these types of data become available, expert and real-world experiences are valuable resources for understanding the ways in which new technologies are being used in leading clinical practices.

## CONCLUSIONS

TVS is a unique technology for the management of cellulite because it includes the ability to discern the contribution (or lack thereof) of fibrous bands to a given cellulite dimple prior to cleaving them as well as confirmation of the release of the septal bands causing a specific dimple following subcision with the device. Together, these features eliminate much of the guesswork inherent in other methods and permit more focal treatment with reproducible resolution of cellulite dimples. Cellulite management is a critical aspect of ensuring patient satisfaction with lower body rejuvenation. TVS is a new technology that shows promise in the management of cellulite and may become an important part of a multimodal approach for nonsurgical management of the buttocks and thighs. It is a welcome addition to the existing armamentarium.
